# Assessment of expression of *interferon γ* (*IFN-G*) gene and its antisense (*IFNG-AS1*) in breast cancer

**DOI:** 10.1186/s12957-018-1508-1

**Published:** 2018-10-18

**Authors:** Hajar Yaghoobi, Hakim Azizi, Vahid Kholghi Oskooei, Mohammad Taheri, Soudeh Ghafouri-Fard

**Affiliations:** 1 Cellular and Molecular Research Center, Basic Health Sciences Institute, Shahrekord University of Medical Sciences, Shahrekord, Iran; 2 Department of Medical Biotechnology, School of Advanced Technologies, Shahrekord University of Medical Sciences, Shahrekord, Iran; 3 Department of Medical Parasitology, School of Medicine, Zabol University of Medical Sciences, Zabol, Iran; 4 Department of Medical Genetics, Shahid Beheshti University of Medical Sciences, Tehran, Iran; 5 Student Research Committee, Shahid Beheshti University of Medical Sciences, Tehran, Iran; 6 Urogenital Stem Cell Research Center, Shahid Beheshti University of Medical Sciences, Tehran, Iran

**Keywords:** IFNG, IFNG-AS1, Breast cancer

## Abstract

**Background:**

The role of long non-coding RNAs has been extensively appreciated in the contexts of cancer. *Interferon γ-antisense RNA1* (*IFNG-AS1*) is an lncRNA located near to IFN-γ-encoding (*IFNG*) gene and regulates expression of *IFNG* in Th1 cells.

**Methods:**

In the present study, we evaluated expression of *IFNG* and *IFNG-AS1* in 108 breast samples including tumoral tissues and their adjacent non-cancerous tissues (ANCTs) using real-time PCR. *IFNG-AS1* was significantly upregulated in tumoral tissues compared with ANCTs (expression ratio = 2.23, *P* = 0.03).

**Results:**

Although the expression of *IFNG* was higher in tumoral tissues compared with ANCTs (relative expression = 1.89), it did not reach the level of significance (*P* = 0.07). *IFNG* expression was significantly higher in HER2-negative tumoral tissues compared with HER2-positive ones (*P* = 0.01) and in grade 1 samples compared with grade 2 ones (*P* = 0.03). No other significant difference was found in expressions of genes between other groups.

**Conclusion:**

Significant strong correlations were detected between expression of *IFNG* and *IFNG-AS1* in both tumoral tissues and ANCTs. The present study provides evidences for participation of *IFNG* and *IFNG-AS1* in the pathogenesis of breast cancer and warrants future studies to elaborate the underlying mechanism.

## Background

Long non-coding RNAs (lncRNAs) are increasingly acknowledged as principal regulators of gene expression in the contexts of both cancer [1] and immunological disorders [2]. Considering the prominent role of immune system in control of carcinogenesis process, lncRNAs with regulatory roles on both immune cells and cancer cells are of particular value as tumor biomarkers or therapeutic targets. *Interferon γ-antisense RNA1* (*IFNG-AS1*) is located near to IFN-γ-encoding (*IFNG*) gene. This lncRNA is regarded as a fundamental checkpoint that participates in IFNG expression in Th1 cells [3]. Targeting immune checkpoint molecules has been suggested as a new approach in cancer treatment. The specific pattern of expression of non-coding RNAs in tumoral tissues and their participation in initial phases of modulation of immune responses have potentiate them as novel candidates for changing the tumor microenvironment [4]. As IFN-based strategies along with immune checkpoint inhibitors are putative therapeutic options for malignancies [4], therapeutic modulation of *IFNG-AS1* expression would exert beneficial effects in cancer patients from diverse aspects. Elevated expression of *IFNG-AS1* lncRNA has been reported in Hashimoto’s thyroiditis (HT) patients in correlation with the proportion of circulating Th1 cells and *IFNG* gene expression [5]. The role of IFN-γ has been documented in both breast cancer pathogenesis and patients’ response to treatments. IFN-γ has been initially recognized for its role in antitumor host immunity which is exerted through induction of Th1 polarization and activation of both cytotoxic T cells (CTLs) and dendritic cells. Nevertheless, in certain conditions, IFN-γ function is in favor of tumor progression which has been documented by the observed negative effect of IFN-γ treatment on patient survival in some clinical trials. The underlying mechanism for such negative effect might be irresponsiveness to IFN-γ, downregulation of the MHC complex, or overexpression of other genes such as programmed cell death 1 ligand 1 (PD-L1) [6]. On the other hand, overexpression of IFN/STAT1-related genes has been suggested as prognostic markers of response to chemotherapy in estrogen receptor (ER) negative breast cancers [7]. More importantly, IFN-γ treatment in conjunction with anti-erbB2/neu mAb has significantly suppressed tumor growth in animal models [8]. In spite of several efforts to evaluate the efficiency of IFN-γ treatment in breast cancer, data regarding expression of *IFNG* gene in breast cancer tissues is scarce. In the present study, we assessed expression of *IFNG* gene and its natural occurring antisense RNA in 108 breast samples including tumoral tissues and their adjacent non-cancerous tissues (ANCTs) using real-time PCR in association with patients clinicopathological characteristics.

## Methods

### Patients

The current study enrolled 54 breast cancer patients. All patients had invasive ductal carcinoma of breast based on the histological examination. All of them have been recently diagnosed as having breast cancer and had no previous chemo/radiotherapy. The patients were admitted to Sina and Farmanieh hospitals over the years 2016–2017. The research protocol was approved by the ethical committee of Shahid Beheshti University of Medical Sciences (IR.SBMU.RETECH.REC.1397.403). All methods were performed in accordance with the relevant guidelines and regulations. Informed written consent was obtained from all patients. Tumoral tissues and ANCTs (0.5 cm × 0.5 cm) were excised from all patients during surgery, transferred in liquid nitrogen to the genetic laboratory of Shahid Beheshti University of Medical Sciences. Tissue samples were assessed by pathologists to endorse the diagnosis.

### Expression analysis

Relative expressions of *IFNG* and *IFNG-AS1* genes were assessed in tumoral tissues and paired ANCTs in the rotor gene 6000 Corbett Real-Time PCR System. Total RNA was extracted from tissue samples using TRIzol™ Reagent (Invitrogen, Carlsbad, CA, USA), and cDNA was synthesized by using RevertAid First Strand cDNA Synthesis Kit (TaKaRa, Japan). Al samples were treated with DNAse I to remove DNA contamination. SYBR Green RT-PCR Master Mix (TaKaRa, Japan) was used for expression analysis of genes. Expressions of genes were normalized to expression of *Beta 2 microglobulin* (*B2M*). The nucleotide sequences of primers are shown in Table 1. All experiments were performed in duplicate.

**Table 1:** The nucleotide sequences of primers used for expression analysis

Gene name	Primer sequence	Primer length	Product length
*B2M*	F: AGATGAGTATGCCTGCCGTG	20	104
	R: CGGCATCTTCAAACCTCCA	19	
*IFNG*	F: GGCAAGGCTATGTGATTACAAGG	23	96
	R:CATCAAGTGAAATAAACACACAACCC	26	
*IFNG-AS1*	F: AGGAAGCTGGGTAATTGAATGC	22	94
	R: CTTAGGAGGAGAATTTTGGGAGAG	24	

### Statistical analysis

Student’s paired *t* test was used for analysis of differences in gene expression between paired samples. The association between clinicopathological data and transcript levels of each gene was assessed using chi-square test. Tukey’s honest significance test was used to assess the difference between mean values of transcript levels between different groups. The efficiency-corrected calculation model was used for assessment of fold changes of expression levels in tumoral tissues vs. ANCTs. The pairwise correlation between relative transcripts levels of *IFNG* and *IFNG-AS1* genes was calculated using the regression model. For all statistical tests, the level of significance was set at *P* < 0.05. The receiver operating characteristic (ROC) curve was designed to assess the properness of gene expression levels for differentiating tumoral vs. ANCTs. The Youden index (j) was used to escalate the difference between sensitivity (true-positive rate) and 1 – specificity (false-positive rate).

## Results

### Elevated levels of *IFNG* and *IFNG-AS1* can be used to identify breast cancer

*IFNG-AS1* was significantly upregulated in tumoral tissues compared with ANCTs (expression ratio = 2.23, *P* = 0.03). Although the expression of *IFNG* was higher in tumoral tissues compared with ANCTs (relative expression = 1.89), it did not reach the level of significance (*P* = 0.07).

**Fig. 1:**
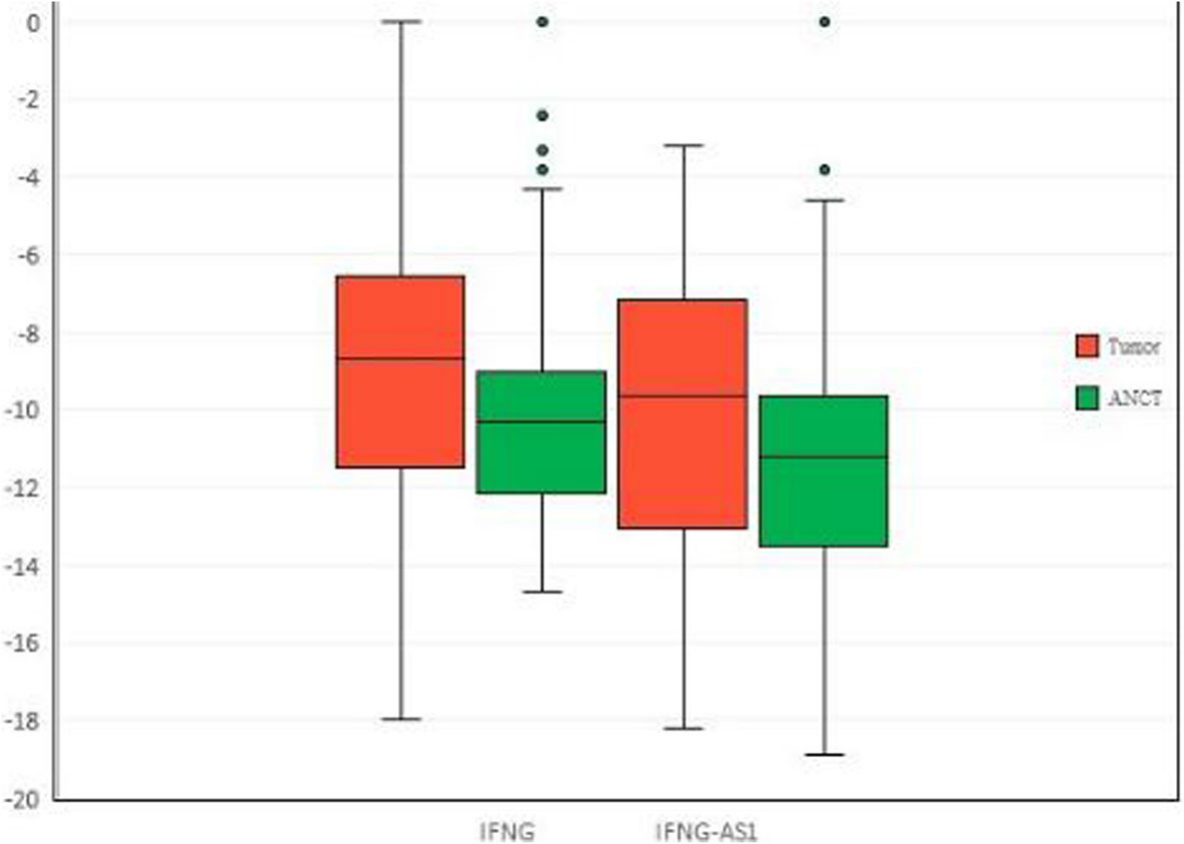
The relative expression of *IFNG* and *IFNG-AS1* in tumoral tissues (*n* = 54) and ANCTs (*n* = 54) as presented by –delta CT values (CT housekeeping - CT target gene) in each set of samples

Figure 1 shows the –delta CT values (CT housekeeping - CT target gene) in tumoral tissues and ANCTs.

**Fig. 2:**
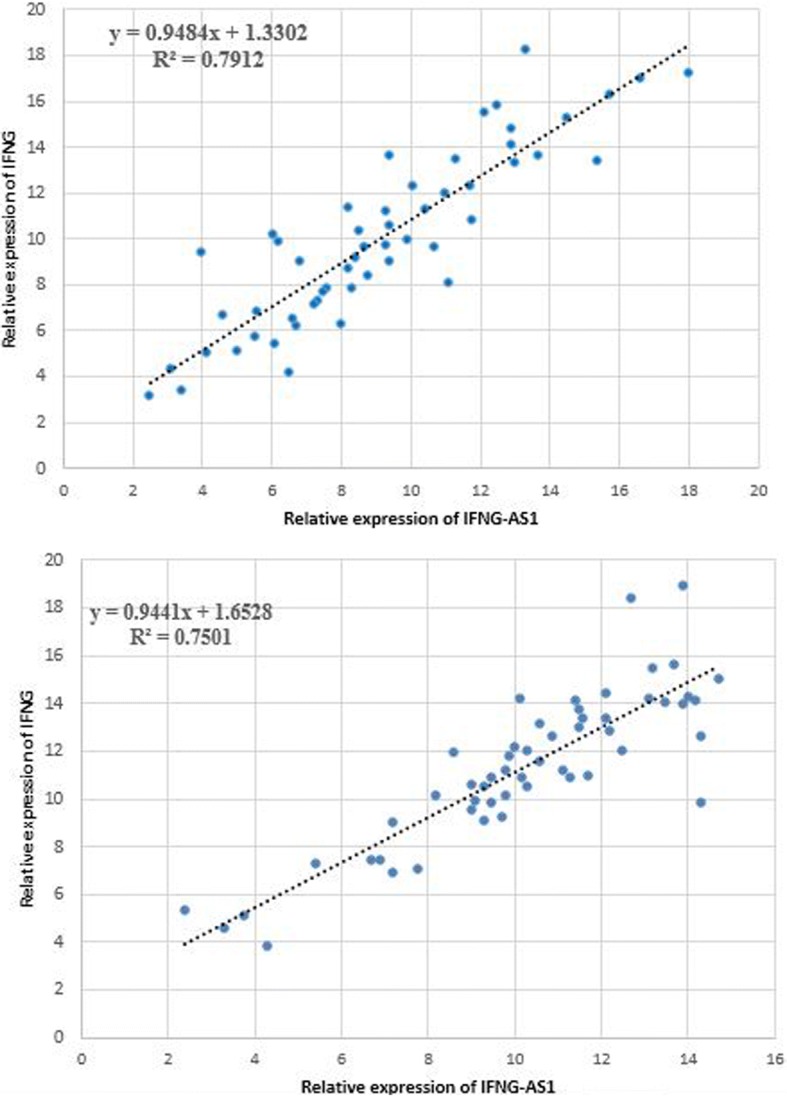
Correlation between relative expressions of *IFNG* and *IFNG-AS1* in tumoral (**a**) and ANCTs (**b**)

Assessment of correlation between expressions of *IFNG* and *IFNG-AS1* genes revealed strong correlations between their expressions in both tumoral tissues and ANCTs (Fig. 2a, b).

### *IFNG* expression status is associated with clinical features of breast cancer

Table 2 shows the summary of demographic and clinicopathological data of study participants which have been gathered from questionnaires and patients’ medical records.

**Table 2:** General demographic data of study participants

Variables	Values
Age (years) (mean ± SD)	51.79 ± 13.54 (29–81)
Menarche age (years) (mean ± SD)	13 ± 1.65 (10–18)
Menopause age (years) (mean ± SD)	44.91 ± 14.91 (38–60)
First pregnancy age (years) (mean ± SD)	18.04 ± 8.36 (14–32)
Breast feeding duration (months) (mean ± SD)	41.62 ± 34.1 (3–120)
Positive family history for cancer (%)	17%
Cancer stage (%)	
I	30.8
II	28.8
III	30.8
IV	9.6
Overall grade (%)	
I	17
II	49
III	34
Mitotic rate (%)	
I	45.2
II	42.9
III	11.9
Tumor size (%)	
< 2 cm	32
≥ 2 cm, < 5 cm	66
≥ 5 cm	2
Estrogen receptor (%)	
Positive	87.8
Negative	12.2
Progesterone receptor (%)	
Positive	77.1
Negative	22.9
Her2/neu expression (%)	
Positive	25
Negative	75
Ki67 expression (%)	
Positive	100
Negative	0

We compared expression level of *IFNG* and *IFNG-AS1* in each tumoral tissue vs. its paired ANCT and classified patients based on these values to upregulation and downregulation groups. *INFG* and *IFNG-AS1* were upregulated in tumoral tissues obtained from 35/54 (64%) and 37/54 (68%) of patients, respectively. Subsequently, we evaluated associations between clinicopathological data and relative expressions of *IFNG* and *IFNG-AS1* genes. No significant associations were found between patients’ clinicopathological data and fold changes of expression of these genes in tumoral tissues compared with ANCTs. Table 3 shows the results of association analysis between relative expressions of genes in tumoral tissues compared with ANCTs and patients’ clinicopathological data.

**Table 3:** The results of association analysis between relative expressions of genes in tumoral tissues compared with ANCTs and patients’ clinicopathological data (up/downregulation of genes was defined based on relative expression of each gene in tumoral tissue compared with the paired ANCT)

	*IFNG* upregulation	*IFNG* downregulation	*P* value	*IFNG-AS1* upregulation	*IFNG-AS1* downregulation	*P* value
Age			0.63			0.5
< 40	8 (67.6%)	3 (27.3%)		9 (81.8%)	2 (18.2%)	
40–50	10 (58.2%)	7 (41.2%)		11 (64.7%)	6 (35.3%)	
51–60	10 (76.9%)	3 (23.1%)		10 (76.9%)	3 (23.1%)	
61–70	5 (62.5%)	3 (37.5%)		5 (62.5%)	3 (37.5%)	
> 71	2 (40%)	3 (60%)		2 (40%)	3 (60%)	
Stage			0.95			0.25
1	9 (56.3%)	7 (43.7%)		10 (62.5%)	6 (37.5%)	
2	10 (66.7%)	5 (33.3%)		9 (60%)	6 (40%)	
3	11 (68.8%)	5 (31.2%)		14 (87.5%)	2 (12.5%)	
4	4 (80%)	1 (20%)		3 (20%)	2 (80%)	
Histological grade			0.87			0.91
1	5 (62.5%)	3 (37.5%)		6 (75%)	2 (25%)	
2	16 (69.6%)	7 (30.4%)		15 (62.5%)	8 (34.8%)	
3	10 (62.5%)	6 (37.5%)		12 (75%)	4 (25%)	
Mitotic rate			0.9			0.64
1	13 (68.4%)	6 (31.6%)		14 (73.7%)	5 (26.3%)	
2	11 (61.1%)	7 (38.9%)		11 (61.1%)	7 (38.9%)	
3	3 (60%)	2 (40%)		4 (80%)	1 (20%)	
Tumor size			0.7			0.67
< 2	9 (56.3%)	7 (43.7%)		12 (75%)	4 (25%)	
2–5	22 (66.7%)	11 (33.3%)		21 (63.6%)	12 (36.4%)	
> 5	1 (100%)	0 (0%)		1 (100%)	0 (0%)	
ER status			0.08			0.65
Positive	26 (60.5%)	17 (39.5%)		29 (67.4%)	14 (32.6%)	
Negative	6 (100%)	0 (0%)		5 (16.7%)	1 (83.3%)	
PR status			0.07			0.46
Positive	22 (59.5%)	15 (40.5%)		25 (67.6%)	12 (32.4%)	
Negative	10 (90.9%)	1 (9.1%)		9 (81.8%)	2 (18.2%)	
HER2 status			0.48			0.27
Positive	7 (58.3%)	5 (41.7%)		7 (58.3%)	5 (41.7%)	
Negative	25 (69.4%)	11 (30.6%)		27 (75%)	9 (25%)	

Next, we compared relative expression of each gene in tumoral samples between clinicopathological-based groups (Table 4). *IFNG* expression was significantly higher in HER2-negative tumoral tissues compared with HER2-positive ones (*P* = 0.01) and in grade 1 samples compared with grade 2 ones (*P* = 0.03). No other significant difference was found in expressions of genes between other groups.

**Table 4:** Comparison of expression levels of *IFNG* and *IFNG-AS1* genes in tumoral tissue of breast cancer patients between clinicopathological-based categories (mean and SD values of (E^CT_B2M_/E^CT_target gene_) are presented)

	*IFNG*	*P* value	*IFNG-AS1*	*P* value
Age				
Pre-menopause vs. post-menopause	0.02 (0.04) vs. 0.03 (0.04)	0.59	0.01 (0.01) vs. 0.01 (0.02)	0.78
ER status				
ER(+) vs. ER(−)	0.03 (0.05) vs. 0.02 (0.01)	0.66	0.01 (0.02) vs. 0.01 (0.01)	0.97
PR status				
PR(+) vs. PR(−)	0.03 (0.05) vs. 0.02 (0.02)	0.74	0.01 (0.02) vs. 0.01 (0.01)	0.92
HER2 status				
HER2(+) vs. HER2(−)	0.009 (0.01) vs. 0.03 (0.05)	0.01	0.01 (0.01) vs. 0.01 (0.02)	0.57
Tumor grade				
Grade 1 vs. 2	0.06 (0.08) vs. 0.01 (0.03)	0.03	0.02 (0.02) vs. 0.01 (0.02)	0.73
Grade 1 vs. 3	0.06 (0.08) vs. 0.02 (0.02)	0.1	0.02 (0.02) vs. 0.01 (0.01)	0.69
Grade 2 vs. 3	0.01 (0.03) vs. 0.02 (0.02)	0.89	0.01 (0.02) vs. 0.01 (0.01)	0.99

### Assessment of the diagnostic value of *IFNG* and *IFNG-AS1* in breast cancer

**Fig. 3:**
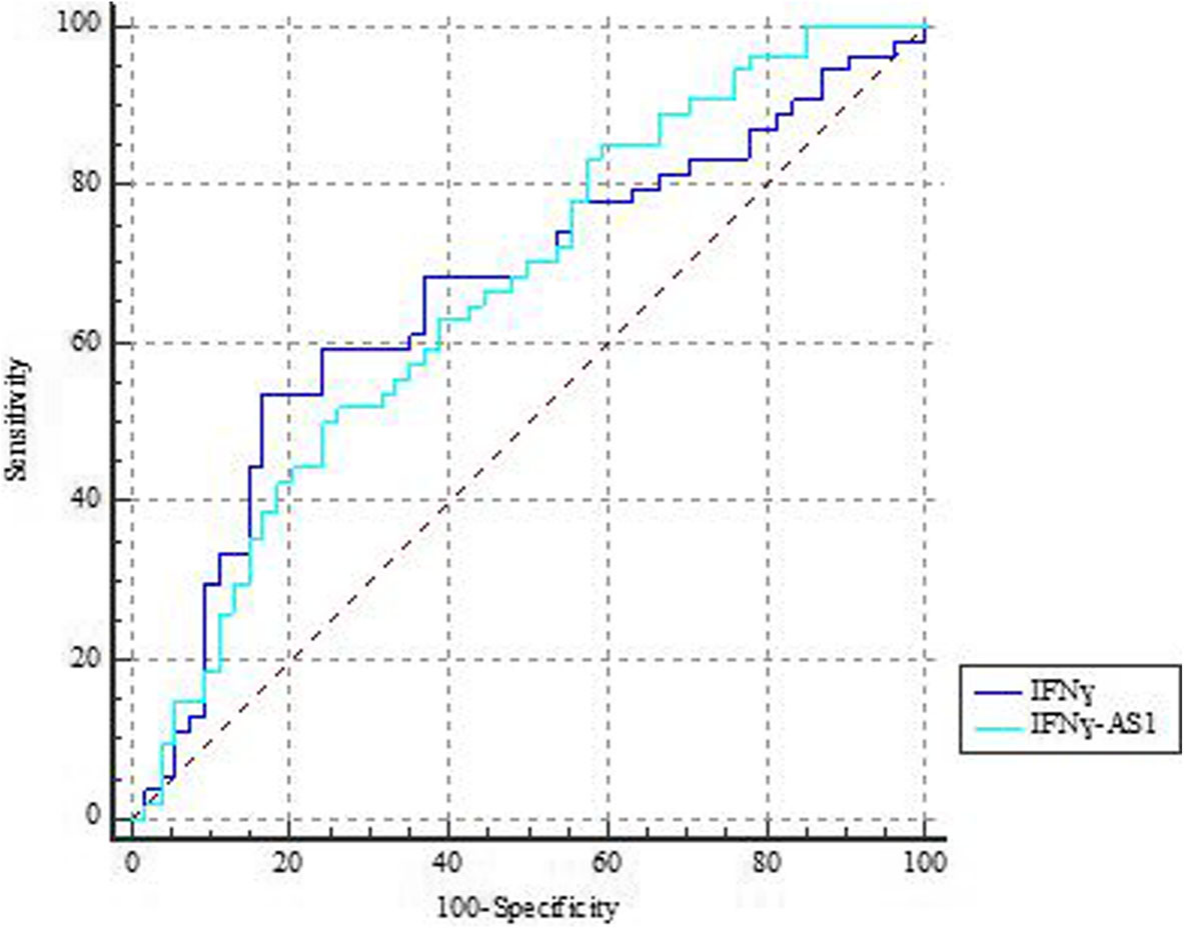
ROC curve for prediction of disease status based on the expression levels of *IFNG* and *IFNG-AS1*

Based on the results of ROC curve analysis *IFNG* and *IFNG-AS1* expressions had 83.3% specificity and 85.2% sensitivity for identification of disease status, respectively. The results of ROC curve analysis are shown in Fig. 3 and Table 5.

**Table 5:** The results of ROC curve analysis

	Estimate criterion	AUC	*J* ^a^	Sensitivity	Specificity	*P* value^b^
*IFNG*	> 0.007	0.664	0.37	53.7	83.3	0.002
*IFNG-AS1*	> 0.0001	0.665	0.25	85.2	40.7	0.001
Combination of both genes	> 0.47	0.665	0.33	61.1	72.2	0.001

Estimate criterion: optimal cutoff point for gene expression

^a^Youden index

^b^Significance level *P* (area = 0.5)

## Discussion

In the present study, we evaluated transcript levels of *IFNG* and *IFNG-AS1* in breast cancer tissues and their paired ANCTs and found significant upregulation of *IFNG-AS1* in tumoral tissues. *IFNG-AS1* has been previously shown to regulate the expression of *IFNG* at both transcriptional and translational level in human CD4+ T cells [5]. Besides, strong positive correlations have been detected between the transcript levels of these two genes in thyroid tissues from HT patients [5]. Our data revealed the similar pattern of correlation between transcript levels of these genes in both tumoral tissues and ANCTs.

We could not find significant difference in expression of *IFNG* between tumoral tissues and ANCTs. García-Tuñón et al. have previously evaluated expression of IFNG in fibrocystic lesions, in situ tumors, and infiltrating tumors of breast and found higher expression of IFNG in in situ carcinoma than in benign and infiltrating tumors. They proposed IFNG as a prospective therapeutic modality in breast cancer [9]. In line with their observation, we found higher levels of *IFNG* in grade 1 samples compared with grade 2 ones. It is possible that tumor cells downregulate expression of *IFNG* as a mechanism for escaping from immune surveillance. We also detected higher *IFNG* expression in HER2-negative tumoral tissues compared with HER2-positive ones. IFN-γ has been previously shown to downregulate expression of HER2 in prostate cancer cells [10]. The existence of similar mechanism in breast cancer cells needs to be assessed. However, the direct effect of IFN-γ on HER2-positive breast cancer cells as reported by Nagai et al. [11] supports a similar function in the context of breast cancer.

Consistent with García-Tuñón et al. [9], we did not find any association between *IFNG* expression and ER/PR status. Mostafa et al. have shown an ERα inhibitory effect on IFN-γ signaling which results in immune escape in ERα-positive breast cancer cells [12]. However, such inhibitory effects are not necessarily exerted on expression of *IFNG* itself. Future studies are needed to assess the effect of estradiol or its receptor on *IFNG* expression.

We observed higher levels of *IFNG-AS1* in breast cancer tissues compared with ANCTs. This finding might be either the cause or the consequence of the tumorigenesis process. Future functional studies are needed to elaborate the consequence of its overexpression in breast tissues. As previous studies have linked its overexpression with autoimmune conditions, it is possible that such overexpression is a compensatory mechanism to conquer immune evasion in tumor microenvironment. Critchley-Thorne et al. have evaluated the effectiveness of IFNG in peripheral blood lymphocytes from breast cancer patients and detected diminished IFN-γ-induced signaling in B cells of these patients in spite of normal signaling in T cells or natural killer cells [13]. Noticeably, no difference has been found within stages II, III, and IV breast cancer patients in this regard [13] which is in accordance with our finding regarding similar expression of the *IFNG* gene in histopathological-based groups. Consequently, there is a cell type-dependent regulatory mechanism for IFN function. So, future studies are needed to elaborate such mechanism in the epithelial tissues obtained from breast tumors to find whether these functional responses are impaired in the cancer tissue. Moreover, the significance of local expression of *IFNG* and *IFNG-AS1* in response to systemic IFN-γ therapy of breast cancer patients must be investigated in imminent researches.

## Conclusion

Apart from functional consequences of dysregulation of *IFNG-AS1* in breast tumor tissues, transcript levels of this gene might be used for diagnosis purposes in the panels of putative biomarkers comprising both coding and non-coding genes. However, based on the results of ROC curve analysis, none of the assessed genes in the present study fulfill the requirements as an individual biomarker as the AUC values of both genes and their combinations were between 0.6 and 0.7 which means the poor accuracy of a diagnostic test.
